# Sinularones A–I, New Cyclopentenone and Butenolide Derivatives from a Marine Soft Coral *Sinularia* sp. and Their Antifouling Activity

**DOI:** 10.3390/md10061331

**Published:** 2012-06-11

**Authors:** Haiyan Shi, Shanjiang Yu, Dong Liu, Leen van Ofwegen, Peter Proksch, Wenhan Lin

**Affiliations:** 1 State Key Laboratory of Natural and Biomimetic Drugs, Peking University, Beijing 100191, China; Email: 775278625@qq.com (H.S.); liudong_1982@126.com (D.L.); 2 China National Center for Biotechnology Development, Beijing 100036, China; Email: yushj@cncbd.org.cn; 3 National Museum of Natural History Naturalis, 2300 RA, Leiden 9515, The Netherlands; Email: ofwegen@naturalis.nnm.nl; 4 Institute of Pharmaceutical Biology and Biotechnology, Heinrich-Heine University, Duesseldorf 40225, Germany; Email: proksch@uni-duesseldorf.de

**Keywords:** soft coral, *Sinularia* sp., sinularones A–I, structural elucidation, antifouling activity

## Abstract

Nine new compounds, namely sinularones A–I (**1**–**9**), characterized as cyclopentenone and butenolide-type analogues, were isolated from a soft coral *Sinularia* sp., together with a known butenolide (**10**). Their structures were elucidated by means of spectroscopic (IR, MS, 1D and 2D NMR, CD) analysis. The absolute configurations were determined on the basis of CD and specific rotation data in association with the computed electronic circular dichroism (ECD) by time dependent density functional theory (TD DFT) at 6-31+G(*d*,*p*)//DFT B3LYP/6-31+G(*d*,*p*) level. Compounds **1**–**2** and **7**–**10** showed potent antifouling activities against the barnacle *Balanus amphitrite*.

## 1. Introduction

**Figure 1:**
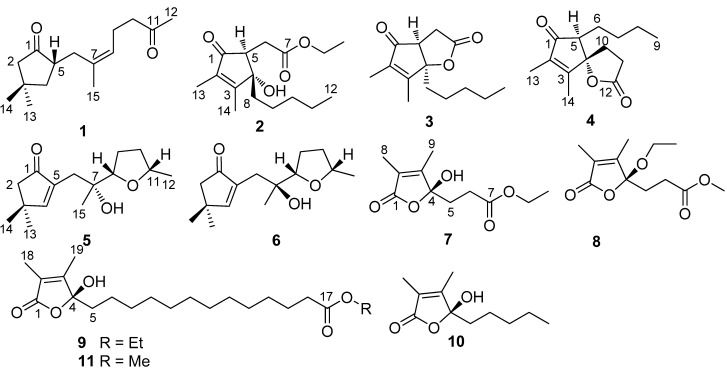
Structures of compounds (**1**–**10**).

The genus *Sinularia* is a dominant biomass with about 100 known species widely distributed in the tropical reef environment [1,2,3,4], presenting a rich array of chemical diversity involving sesquiterpenes, diterpenes, polyhydroxylated steroids, and polyamine compounds. Several metabolites derived from *Sinularia* play an active role in chemical defense [5,6], and display a range of biological activities, such as antimicrobial [7], anti-inflammatory [8], and cytotoxic [9,10]. In the course of our search for bioactive natural products from marine invertebrates inhabited in the South China Sea, an octocoral *Sinularia* sp. was collected off Hainan Island. Chemical examination of the EtOAc soluble fraction of this specimen resulted in the isolation of the new cyclopentenone derivatives (**1**–**6**) and furanones (**7**–**9**), along with the known butenolide (**10**) (Figure 1). This paper reports the structural elucidation of the new compounds and the evaluation of their antifouling activities.

## 2. Results and Discussion

**Figure 2:**
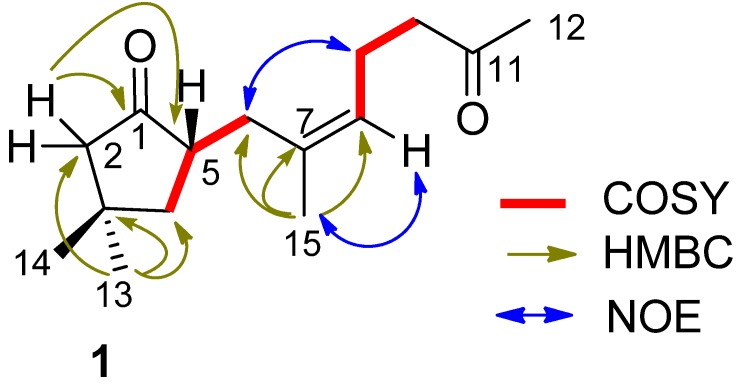
Key COSY, HMBC and NOE relationships of (**1**).

**Figure 3:**
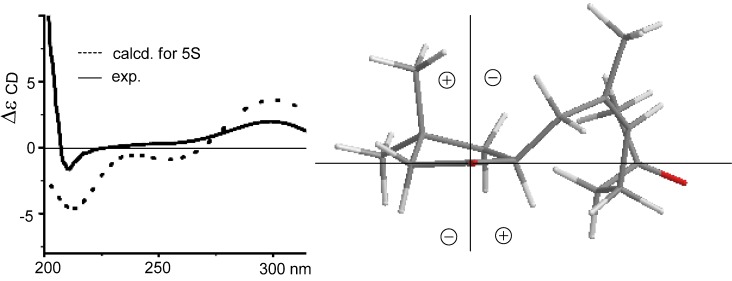
CD, electronic circular dichroism (ECD) and octant rule of (**1**).

Sinularone A (**1**) has a molecular formula of C_15_H_24_O_2_ as established by the HRESIMS (*m/z* 259.1674 [M + Na]^+^, calcd. 259.1674) and NMR data, indicating 4° unsaturation. The IR absorptions at 1738 and 1717 cm^−1^ suggested the presence of carbonyl groups. The ^1^H NMR spectrum exhibited the signals for a single olefinic proton at *δ*_H_ 5.12 (1H, t, *J* = 7.2 Hz, H-8), four methyl singlets at *δ*_H_ 1.01 (3H, s, H_3_-13), 1.13 (3H, s, H_3_-14), 1.61 (3H, s, H_3_-15) and 2.07 (3H, s, H_3_-12), together with 11 aliphatic protons. APT spectra displayed 15 carbon resonances, involving two ketones at *δ*_C_ 220.0 (C-1) and 208.5 (C-11), and two olefinic carbons at *δ*_C_ 125.7 (CH, C-8) and 133.9 (C, C-7). The proton signals and their associated carbons were assigned by HMQC. The COSY cross-peaks allowed to establish the proton-proton spin systems from C-4 to C-6 and from C-8 to C-10, while the HMBC interactions from the olefinic H_3_-15 to C-6 (*δ*_C_ 32.3), C-7, and C-8, and from H_3_-12 to C-11 and C-10 (*δ*_C_ 43.4) led to the connectivity of the subunits to form a linear chain defined as a 7-methylhept-7-en-11-one group (Figure 2). Additional HMBC interactions from the geminal protons H_α__,β_-2 (*δ*_H_ 2.08, 2.16) to C-3 (*δ*_C_ 34.0), C-4 (*δ*_C_ 43.5), C-5 (*δ*_C_ 46.7) and C-1, along with the correlations from both H_3_-13 and H_3_-14 to C-2 (*δ*_C_ 52.8), C-3, and C-4, and from H-5 (*δ*_H_ 2.49, m) to C-1 and C-3 indicated the presence of a 3,3-dimethylcyclopentanone nucleus. Subsequently, the linear side chain was determined to be linked to the nucleus at C-5 on the basis of the COSY relationships in addition to the HMBC interactions from H_2_-4 (*δ*_H_ 1.41, 1.84) to C-6 and from H_2_-6 to C-1, C-4, and C-5. The geometry of Δ^7^ was assigned to 7*Z* according to the NOE interaction between H_3_-15 and H-8 in addition to the chemical shift of C-15 (*δ*_C_ 23.5), higher than 20 ppm [11]. In regard to the configuration of the stereogenic center C-5, the CD spectrum showed a negative Cotton effect at 212 nm for *n*–π* transition in agreement with the electronic circular dichroism (ECD) data which was calculated for 5*S* configuration by time dependent density functional theory (TD DFT) at 6-31+G(*d*,*p*)//DFT B3LYP/6-31+G(*d*,*p*) level [12]. This assignment was also supported by the octant rule which allowed the side chain to be located at the negative CD region (Figure 3) [13]. 

**Figure 4:**
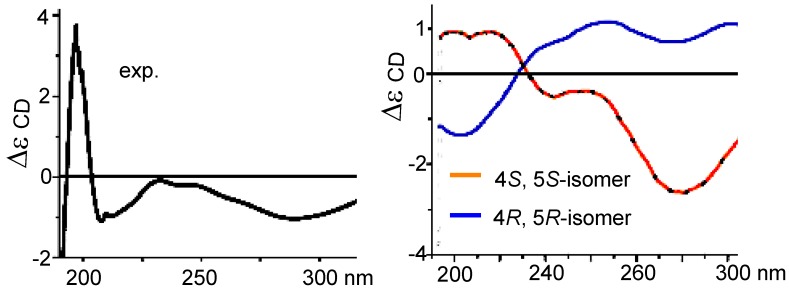
CD and ECD spectra of (**2**).

Sinularone B (**2**) has a molecular formula of C_16_H_26_O_4_ as determined by the HRESIMS (*m/z* 305.1732 [M + Na]^+^, calcd. 305.1729) and NMR data, indicating 4° unsaturation. The IR absorptions at 3489, 1737, 1707 and 1653 cm^−1^ suggested the presence of hydroxy, carbonyl, and oleﬁnic groups. The ^1^H NMR spectrum exhibited two terminal methyls appearing as doublets at *δ*_H_ 0.80 (3H, t, *J* = 7.0 Hz, H_3_-12) and 1.21 (3H, t, *J* = 7.1 Hz, H_3_-1′), and two olefinic methyl singlets at *δ*_H_ 1.62 (3H, s, H_3_-13) and 1.90 (3H, s, H_3_-14), in addition to 13 aliphatic protons (Table 1). APT spectrum displayed a total of 16 carbon resonances, of which two carbonyl carbons, two olefinic carbons, an oxygen-bearing sp^3^ carbon, and an oxymethylene were observed (Table 2). The presence of a *n*-pentyl unit was recognized by the COSY and HMBC relationships to establish a side chain from C-8 to C-12, while an ethoxy group was ascribed to the COSY coupling between H_3_-1′ and *δ*_H_ 4.10 (2H, q, *J* = 7.1 Hz, H_2_-2′). In HMBC spectrum, the interactions from H_3_-13 to the carbons at *δ*_C_ 203.8 (C-1), 134.3 (C-2), and 170.0 (C-3), and from H_3_-14 to C-2, C-3, and C-4 (*δ*_C_ 80.1), in addition to the interaction from a methine proton *δ*_H_ 2.89 (1H, dd, *J* = 5.6, 8.4, H-5) to C-1, C-2, C-3, and C-4, conducted to establish a 2,3-dimethylcyclopent-2-enone nucleus. The COSY coupling between H-5 and the methylene protons H_2_-6 at *δ*_H_ 2.44 (1H, dd, *J* = 5.6, 16.0 Hz) and 2.35 (1H, dd, *J* = 8.4, 16.0 Hz) in association with the HMBC interaction from the carbonyl carbon (*δ*_C_ 172.5, C-7) to H-5, H_2_-6, and the oxymethylene H_2_-2′, ascertained an ethylacetate unit linked to C-5 (*δ*_C_ 55.3). The oxygenated quaternary carbon C-4 was determined to be co-positioned by a *n*-pentyl unit and a hydroxy group on the basis of the HMBC interactions between H-5 and the methylene carbon C-8 (*δ*_C_ 35.9), and in turn from H_2_-8 (*δ*_H_ 1.54, 1.58) to C-3, C-4, and C-5, in addition to a D_2_O exchangeable proton (*δ*_H_ 5.28, s) showing HMBC relationship with C-4. The NOE interaction between H_2_-6 and H_2_-8 (*δ*_H_ 1.54, 1.58) was indicative of their *cis* relationship. Thus, the relative configurations of the stereogenic centers were depicted to be 4S* and 5S*. The measured CD curve of **2** was closely similar to the calculated ECD for 4*S*,5*S*-isomer in opposite to the data for 4*R*,5*R*-isomer (Figure 4), indicating **2** to be in agreement with 4*S* and 5*S*.

The NMR spectroscopic data of sinularone C (**3**) were closely related to those of **2**, except for the absence of the signals for an ethoxy group. Analysis of 1D and 2D NMR (COSY, HMQC and HMBC) disclosed a 2,3-dimethylcyclopent-2-enone nucleus, and a *n*-pentyl group being linked to the nucleus at C-4, showing the partial structure to be the same as that of **2**. The COSY cross-peaks between H-5 (*δ*_H_ 3.11) and H_2_-6 (*δ*_H_ 2.49, 3.08) and the HMBC interactions of these protons with a carbonyl carbon C-7 (*δ*_C_ 174.9) and the ketone C-1 (*δ*_C_ 204.9) allowed to link C-6 of an acetyl unit to cyclopent-2-enone nucleus at C-5. In addition, a quaternary carbon observed at *δ*_C_ 92.4 was assigned to C-4 on the basis of the HMBC interactions of C-4 to H-5 and H_2_-6. The molecular formula (C_1__4_H_2__0_O_3_) (HRESIMS *m/z* 259.1315 [M + Na]^+^) of **3** showing a C_2_H_6_O unit less than that of **2** and requiring 5° unsaturation, in association with the obvious downfield shifted C-4 in comparison with that of **2**, allowed to connect C-4 and C-7 to form a γ-lactone, which was fused to the cyclopentenone ring across C-4 and C-5. The observed NOE interactions from H-5 to H_2_-8 (*δ*_H_ 1.79, 1.97) and H_2_-9 (*δ*_H_ 1.10, 1.16) indicated H-5 to be oriented in the same face as *n*-pentyl group. This assignment was supported by the irradiation of H_2_-8 causing the NOE enhancement of H-5 (1.7%, 1.2%). Compound **3** is likely a precursor to generate **2** by ethoxylation. Thus, the absolute configuration of C-5 in **3** is suggested to be the same as that of **2**. Accordingly, the chiral centers of **3** were assumed to be 4*R* and 5*S*.

**Table 1:** ^1^H NMR data of sinulactones A–H (**1**–**8**) (500 MHz, *δ*_H_, *J* in Hz).

No.	1 ^a^	2 ^a^	3 ^a^	4 ^a^	5 ^a^	6 ^a^	7 ^a^	8 ^b^
2	2.08 d (16.4)				2.18 s	2.18 d (18.0)		
	2.16 d (16.4)				2.18 s	2.22 d (18.0)		
4	1.41 dd (11.5, 12.4)				7.35 s	7.35 s		
	1.84 dd (8.4, 12.4)							
5	2.49 m	2.89 dd (5.6, 8.4)	3.11 dd (5.5, 10.2)	2.55 t (6.5)			1.91 ddd (6.0, 6.0, 17.0)	1.97 ddd (7.8, 7.8, 15.5)
							2.32 ddd (6.0, 8.0, 17.0)	2.22 m
6	2.06 m	2.35 dd (8.4, 16.0)	2.49 dd (10.2, 15.5)	1.45 m	2.09 d (13.9)	2.21 s	2.49 ddd (6.0, 6.0, 18.0)	2.24 m
	2.29 dd (4.2, 13.4)	2.44 dd (5.6, 16.0)	3.08 dd (5.5, 15.5)	1.67 m	2.27 d (13.9)	2.21 s	2.78 ddd (6.0, 8.0, 18.0)	2.27 m
7				1.30 m				
				1.46 m				
8	5.12 t (7.2)	1.54 ddd (4.0, 11.0, 12.0)	1.79 ddd (4.7, 12.1, 13.5)	1.30 m	3.57 t (7.2)	3.56 dd (7.4, 7.4)	1.80 s	1.77 s
		1.58 ddd (5.5, 11.0, 12.0)	1.97 ddd (4.5, 12.1, 13.5)	1.32 m				
9	2.16 ddt (7.2, 7.4, 12.0)	0.53 m	1.10 m	0.88 t (7.2)	1.75 m	1.71 m	1.96 s	1.87 s
	2.19 ddt (7.2, 7.4, 12.0)	0.66 m	1.16 m		1.80 m	1.78 m		
10	2.44 dd (7.4, 7.4)	1.15 m	1.29 m	2.16 ddd (7.7, 10.6, 13.9)	1.27 m	1.30 m		
				2.23 ddd (6.2, 10.3, 13.9)	1.87 m	1.87 m		
11		1.18 m	1.29 m	2.72 ddd (7.7, 10.3, 18.4)	3.83 m	3.86 m		
				2.78 ddd (6.2, 10.6, 18.4)				
12	2.07 s	0.80 t (7.0)	0.86 t (7.1)		1.13 d (6.0)	1.14 d (6.0)		
13	1.01 s	1.62 s	1.67 s	1.65 s	1.15 s	1.15 s		
14	1.13 s	1.90 s	2.02 s	1.97 s	1.15 s	1.15 s		
15	1.61 s				0.87 s	0.84 s		
EtO		4.10 q (7.1)					4.17 q (7.1)	3.13 dq (7.0, 12.0)
		1.21 t (7.1)					1.27 t (7.1)	3.24 dq (7.0, 12.0)
								1.09 t (7.0)
								3.58 s
OH-4		5.28 s					5.26 s	
OH-7					4.16 s	4.16 s		

^a^ In DMSO-*d*_6_, ^b^ In CDCl_3_.

**Table 2:** ^13^C NMR data of sinulactones A−H (**1**−**8**) (125 MHz, *δ*_C_, mult.).

No.	1 ^a^	2 ^a^	3 ^a^	4 ^a^	5 ^a^	6 ^a^	7 ^a^	8 ^b^
1	220.0 C	203.8 C	204.9 C	203.4 C	209.4 C	209.4 C	171.8 C	171.1 C
2	52.8 CH_2_	134.3 C	138.7 C	136.4 C	49.8 CH_2_	49.8 CH_2_	124.7 C	127.1 C
3	34.0 C	170.0 C	166.7 C	164.9 C	38.9 C	38.9 C	158.2 C	156.7 C
4	43.5 CH_2_	80.1 C	92.4 C	91.5 C	170.8 C	170.7 C	105.5 C	109.1 C
5	46.7 CH	55.3 CH	46.4 CH	54.5 CH	138.8 C	138.9 C	31.3 CH_2_	31.3 CH_2_
6	32.3 CH_2_	29.6 CH_2_	32.3 CH_2_	24.8 CH_2_	33.5 CH_2_	32.2 CH_2_	28.9 CH_2_	28.0 CH_2_
7	133.9 C	172.5 C	174.9 C	29.3 CH_2_	72.5 C	72.3 C	174.8 C	172.9 C
8	125.7 CH	35.9 CH_2_	34.0 CH_2_	22.2 CH_2_	85.2 CH	85.2 CH	8.4 CH_3_	8.6 CH_3_
9	22.3 CH_2_	24.6 CH_2_	23.0 CH_2_	13.7 CH_3_	26.2 CH_2_	26.1 CH_2_	10.6 CH_3_	11.0 CH_3_
10	43.4 CH_2_	31.9 CH_2_	31.7 CH_2_	25.3 CH_2_	33.3 CH_2_	33.2 CH_2_		
11	208.5 C	22.3 CH_2_	22.3 CH_2_	28.7 CH_2_	75.3 CH	75.2 CH		
12	30.2 CH_3_	14.3 CH_3_	14.3 CH_3_	176.1 C	21.3 CH_3_	21.4 CH_3_		
13	28.1 CH_3_	8.0 CH_3_	8.3 CH_3_	7.8 CH_3_	28.6 CH_3_	28.5 CH_3_		
14	29.9 CH_3_	11.6 CH_3_	12.3 CH_3_	10.6 CH_3_	28.4 CH_3_	28.5 CH_3_		
15	23.5 CH_3_				21.9 CH_3_	23.4 CH_3_		
16								
17								
18								
EtO		14.5 CH_3_					14.1 CH_3_	15.4 CH_3_
		60.5 CH_2_					61.5 CH_2_	58.4 CH_2_
MeO								51.9 CH_3_

^a^ In DMSO-*d*_6_, ^b^ In CDCl_3_.

**Figure 5:**
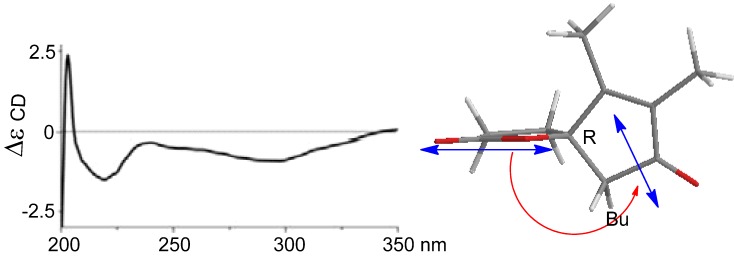
Cotton effect of (**4**).

The molecular formula (C_14_H_20_O_3_) of sinularone D (**4**) was determined to be the same as that of **3** on the basis of the HRESIMS data (*m/z* 259.1313 [M + Na]^+^, calcd. 259.1310) and NMR data. The IR absorptions at 1778, 1710 and 1657 cm^−1^ were characteristic of lactone, ketone, and olefinic groups. Comparison of the NMR data revealed the resonances of **4** in respect to those of a 2,3-dimethylcyclopent-2-enone nucleus (Table 1 and Table 2) compatible to the data of **3**. However, the COSY and HMBC relationships revealed a *n*-butyl group to replace a *n*-pentyl group of **3**. This unit was determined to be linked to C-5 (*δ*_C_ 54.5) as evident from the COSY correlation between H_2_-6 (*δ*_H_ 1.45, 1.67) and H-5 (*δ*_H_ 2.55, t, *J* = 6.5 Hz) in association with the HMBC interactions from H_2_-6 to C-1 (*δ*_C_ 203.4). The remaining resonances included two methylenes and a carbonyl carbon (*δ*_C_ 176.1, C-12). The COSY correlation between H_2_-10 (*δ*_H_ 2.16, 2.23) and H_2_-11 (*δ*_H_ 2.72, 2.78) and the HMBC interactions from H_2_-10 to C-12 and from H_2_-11 to C-4 (*δ*_C_ 91.5, C), in association with the molecular formula requiring 5° unsaturation, conducted the quaternary carbon C-4 to be located by a γ-lactone. The NOE interaction between H_2_-6 and H_2_-10 reflected the same orientation of H-5 and the heterocyclic atom of lactone. In regard to the absolute configuration of the *spiro* carbon C-4, we extended the CD method originally used for the chiral center of allylic amines [14]. The negative Cotton effect at 222 nm belonging to the π–π* charge-transfer transition polarized by the lactone carbonyl group and the enone unit, correlated with anti-clockwise screw (Figure 5). Therefore, the sign of the Cotton effect reflected a 4*R* configuration. In combination with NOE data, C-5 was thus assigned to *S* configuration. 

The NMR spectroscopic data of **5** and **6** are very similar, while HRESIMS data revealed both compounds shared the same molecular formula (C_15_H_24_O_3_), indicating 4° unsaturation. APT spectrum of **5** displayed 15 carbon resonances, involving one ketone (*δ*_C_ 209.4, C-1), two olefinic carbons (*δ*_C_ 138.8, C-5; 170.8, C-4), two oxymethines at *δ*_C_ 85.2 (C-8) and 75.3 (C-11) and four methyls (*δ*_C_ 21.3, 21.9, 28.4, 28.6). The observation of HMBC interactions from the isolated methylene protons at *δ*_H_ 2.18 (2H, s, H_2_-2) to C-1, C-3 (*δ*_C_ 38.9, C), C-4, and C-5 and from H-4 (*δ*_H_ 7.35, s) to C-1, C-2, and C-3, in addition to the methyl singlets at *δ*_H_ 1.15 (6H, s, H_3_-13 and H_3_-14) to C-2, C-3, and C-4, ascertained the presence of 5-substituted 3,3-dimethylcyclopent-4-enone. In regard to the remaining resonances, the COSY correlations extended a moiety from C-8 to C-12, in which C-8 and C-11 were oxygenated. Additionally, the HMBC interactions from H_3_-15 (*δ*_H_ 0.87, s) to the methylene carbon C-6 (*δ*_C_ 33.5), quaternary carbon C-7 (*δ*_C_ 72.5) and C-8 and from H-8 (*δ*_H_ 3.57, t, *J* = 7.2 Hz) to C-11 indicated the formation of an ether bridge across C-8 and C-11, while C-7 was co-positioned by a methyl and a hydroxy groups. The side chain was linked to C-5 as evident from the HMBC relationships of H_2_-6 (*δ*_H_ 2.09, 2.27) with C-1, C-5, and C-4. Based on the NOE interaction between H-8 and H-11, a *cis*-geometry of the 8,11-epoxy bonding was assigned. Thus, the relative configurations were suggested to be 8S* and 11S*. 

Analysis of 2D NMR data revealed sinularone F (**6**) to be a stereoisomer of **5**. Both compounds showed the NOE interaction between H-8 and H-11, indicating them to be oriented in the same face toward tetrahydrofuran ring. The major difference was found by the chemical shifts at C-6 and C-15 (Table 2), implying **6** to be a C-7 epimer of **5** rather than an enantiomer. Since the calculated ECD data could not provide confidential evidence to judge the configurations, the calculation for specific rotation and ^13^C NMR data was performed. The conformations with relative energies from 0 to 2.5 kcal/mol were used in optical rotation computations at B3LYP/6-311+G(2*d*,*p*) level, while Boltzmann statistics were used for rotation computations of all conformations. The computed specific rotation [15] for 7*S*,8*R*,11*R*-isomer is −16.9 and −27.5 for 7*R*,8*R*,11*R*-isomer. These data are in opposite sign to the experimental data that of **5** ([α]_D_^23^ +18.0) and **6** ([α]_D_^23^ +22.6), indicating **5** and **6** to be the enantiomers of the calculated isomers. These assignments were also supported by the relative shift errors of the experimental ^13^C NMR data of **6** and **5** that were in accordance with the error distribution calculated at B3LYP/6-311+G(2*d*,*p*) level (Table 3) [16]. Thus, the absolute configurations of **5** were in agreement with 7*R*,8*S*,11*S*, whereas those of **6** were assigned to 7*S*,8*S*,11*S*.

**Table 3:** The error patents of ^13^C NMR data of **6** and **5** in experiments and computations.

Position	6	5	Δ*δ*_6−5_	6 *	5 *	Δ*δ*_6−5_
1	209.4	209.4	0.0	206.0	205.8	0.2
2	49.8	49.8	0.0	49.2	49.2	0.0
3	38.9	38.9	0.0	41.9	42.0	−0.1
4	170.7	170.8	−0.1	171.2	171.1	0.1
5	138.9	138.8	0.1	142.8	142.5	0.3
6	32.2	33.5	−1.3	32.7	39.8	−7.1
7	72.3	72.5	−0.2	72.4	72.7	−0.3
8	85.2	85.2	0.0	84.8	84.1	0.7
9	26.1	26.2	−0.1	27.2	27.4	−0.2
10	33.2	33.3	−0.1	34.3	34.7	−0.4
11	75.2	75.3	−0.1	75.7	75.8	−0.1
12	21.4	21.3	0.1	18.5	18.6	−0.1
13	28.5	28.6	−0.1	26.2	26.4	−0.2
14	28.5	28.4	0.1	26.2	26.2	0.0
15	23.4	21.9	1.5	24.4	17.4	7.0

**6** *: calculated for 7*S*,8*S*,11*S*-isomer; 5*: calculated for 7*R*,8*S*,11*S*-isomer.

Sinularone G (**7**) has a molecular formula of C_1__1_H_1__6_O_5_ as determined by HRESIMS data (*m/z* 251.0886 [M + Na]^+^, calcd. 251.0895), requiring 4° unsaturation. The ^1^H NMR exhibited two olefinic methyl singlets at *δ*_H_ 1.80 (3H, s, H_3_-8) and 1.96 (3H, s, H_3_-9) and a methyl triplet (*δ*_H_ 1.27, H_3_-1′), while ^13^C NMR involved two carbonyl carbons at *δ*_C_ 171.8 (C-1) and 174.8 (C-7), two olefinic carbons at *δ*_C_ 124.7 (C-2) and 158.2 (C-3), and an acetal carbon at *δ*_C_ 105.5 (C-4). The HMBC interactions of H_3_-8 to C-1, C-2, and C-3 and from H_3_-9 to C-2, C-3, and C-4 disclosed a α,β-unsaturated 2,3-dimethyl-γ-lactone, the same as that of a known butenolide [17]. In addition, an ethylpropanoate was recognized by the COSY relationships between two vicinal methylenes along with a methyl triplet H_3_-1′ coupled to the oxymethylene at *δ*_H_ 4.17 (H_2_-2′), in combination with the HMBC interactions from C-7 to the protons of H_2_-6 (*δ*_H_ 2.49, 2.78), H_2_-5 (*δ*_H_ 1.91, 2.32), and the oxymethylene (*δ*_C_ 61.5). The positive specific rotation ([α]_D_ +4.03°) and the similar Cotton effect in comparison with those of a known butenolide (**11**) supposed C-4 to be 4*S* configuration [11,17].

The NMR data of sinularone H (**8**) were similar to those of **7** with the exception of the presence of an additional methoxy group. Examination of the HMBC cross-peaks afforded the interactions between the carbonyl carbon (*δ*_C_ 172.9, C-7) and the methoxy protons (*δ*_H_ 3.58, s) and between the acetal carbon (*δ*_C_ 109.1, C-4) and the oxymethylene (*δ*_H_ 3.13, 3.24), requiring the formation of a methyl ester, while the ethoxy group was substituted to C-4. The absolute configuration of C-4 was supposed to be the same as that of **7** on the basis of similar specific rotation and CD data.

The NMR data of sinularone I (**9**) were mostly identical to those of a known butenolide (**11**) [17]. The distinction was attributed to the presence of an ethyl ester to replace a methyl ester of the known analogue, as evident from the molecular weight of **9** (C_21_H_36_O_5_) to be 14 amu more than that of the latter, and the presence of an ethoxy group in its NMR spectra. The absolute configuration was determined to be 4*S* on the basis of the similar specific rotation and Cotton effect as those of **8** and the known analogues [17].

In addition, the spectroscopic data and the specific rotation indicated the butenolide **10** to be identical to (*S*)-4-hydroxy-2,3-dimethyl-4-pentyl-γ-lactone, isolated from the fruiting bodies of a fungus [11].

All compounds were tested for their cytotoxicity against a panel of tumor cell lines including human ovarian carcinoma A2780, human lung adenocarcinoma A549, human gastric carcinoma BGC823, human hepatoma Bel7402, and human colonic carcinoma HCT-8. However, they showed weak inhibitory activity with IC_50_ > 10 μg/mL. In order to detect whether these compounds play a role for ecological functions, the test for antifouling activity against the larvae of the barnacle *Balanus amphitrite* was performed [18,19]. The bioassay results revealed compounds **1**–**2**, **7**–**10** showed potent inhibition with the EC_50_ values (Table 4) lower than the standard requirement (EC_50_ < 25 μg/mL) in regard to the efficacy level of natural antifouling agents as established by the US navy program [20]. However, compounds **3**–**6** showed weak inhibition with EC_50_ > 50 μg/mL. In addition, the bioactive compounds (**1**–**2**, **7**–**10**)showed weak toxicity against the barnacle with LC_50_ > 50 μg/mL. A primary discussion of structure-activity relationship implied that α,β-unsaturated 2,3-dimethyl-γ-lactone is a functional unit for anti-barnacle. Among the active compounds, **10** is the most active, suggesting it to be a promising candidate as a nontoxic natural antifouling agent.

**Table 4:** Antifouling activity of compounds against the larvae of barnacle B. amphitrite *.

Compounds	*Balanus amphitrite* Larvae		
	EC_50_ (µg/mL)	LC_50_ (µg/mL)	LC_50_/EC_50_
**1**	13.86	>50	>3.61
**2**	23.50	>50	>2.13
**7**	18.65	>50	>2.69
**8**	21.39	>50	>2.34
**9**	12.58	>50	>3.97
**10**	3.84	>50	>13.02

* EC_50_ represents the concentration of a compound where 50% of larval population was inhibited to settle compared to control, while LC_50_ represents the concentration of a compound required to kill 50% of larvae of a tested population.

## 3. Experimental Section

### 3.1. General

Optical rotations were measured on a Perkin-Elmer 243B polarimeter. IR spectra were determined on a Thermo Nicolet Nexus 470 FTIR spectrometer. CD spectra were measured using J-810-150s spectropolarimeter (Jasco, Darmstadt, Germany). ^1^H and ^13^C NMR and 2D NMR spectra were recorded on a Bruker Avance 600 MHz and Bruker Avance 500 MHz using TMS as an internal standard. HRESIMS data were obtained on a LTQ Orbitrap XL instrument. HPLC was performed with a C_18_ packed column (250 × 10 nm) and using a DAD detector.

### 3.2. Animal Material

The soft coral *Sinularia* sp. was collected from the inner coral reef at a depth of around 8 m in Hainan Island of China, in May 2004, and the samples were frozen immediately after collection. The specimen was identified by Leen van Ofwegen (National Museum of National History Naturalis). The voucher specimens (HSF-15) are deposited at the State Key Laboratory of Natural and Biomimetic Drugs, Peking University, China.

### 3.3. Extraction and Isolation

The soft coral *Sinularia* sp. (3.7 kg) was homogenized, and then extracted with 95% EtOH (5 L × 3). The EtOH extract (102.5 g) was partitioned between H_2_O (400 mL) and EtOAc (200 mL). The EtOAc fraction (10.0 g) was subjected to column chromatography (3.5 × 25 cm) using 160–200 mesh Si gel (120 g) and was eluted with a gradient of petroleum ether (PE)/acetone (20:1, 10:1, 1:1) to obtain nine fractions (F1–F9). F3 (260 mg) was fractionated on an ODS column (C_18_, 2.0 × 25 cm) and eluted with a gradient of MeOH/H_2_O (75%–100%) to collect three portions PA–PC. Portion PB (85% MeOH, 180 mg) were then subjected to a Sephadex LH-20 column (1.5 × 35 cm) eluting with acetone to give **2** (7.0 mg), **3** (11.7 mg), and **4** (2.6 mg). Portion PA (75% MeOH, 70 mg) was subjected to semi-preparative HPLC (ODS, 5 μm, 2 × 25 cm) with 50% MeOH as a mobile phase to obtain **5** (1.5 mg), **6** (1.4 mg), **7** (2.4 mg), and **10** (3.2 mg). Compounds **1** (14.9 mg), **8**(3.5 mg) and **9** (3.4 mg) were isolated from F4 (300 mg) using Sephadex LH-20 column (1.5 × 35 cm) and semi-preparative RP-18 HPLC (5 μm, 2 × 25 cm) with 45% MeOH as a mobile phase.

### 3.4. Computational Calculation

The computational ECD, specific rotation, and ^13^C NMR calculations were performed by the B3LYP functional and a generic basis set, employ the 6-311+G(*d*,*p*) basis set [21,22]. This generic basis set has been shown to be effective, both efficient and reliable, in predicting structural and reactivity properties for homogeneous systems. All calculations are performed with the Gaussian 03 package with tight self-consistent field convergence and ultrafine integration grids.

### 3.5. Larval Settlement Bioassays

Adults of the barnacle *Balanus amphitrite* Darwin were exposed to air for more than 6 h, and then were placed in a container filled with fresh 0.22 µm filtered sea water (FSW) to release nauplii. The collected nauplii were reared to cyprid stage according to the method described by Thiyagarajan *et al*. [23]. When kept at 26–28 °C and fed with *Chaetoceros gracilis*, larvae developed to cyprids within four days. Fresh cyprids were used in the tests.

### 3.6. Cytotoxic Assay

The cytotoxic properties of the isolated compounds were tested in vitro using human tumor cell lines including human ovarian carcinoma cell line A2780, human lung adenocarcinoma epithelial cell line A549, human gastric carcinoma cell line BGC823, human hepatoma cell line Bel7402, and human colonic carcinoma cell line HCT-8 by MTT method.

Sinularone A (**1**): obtained as colorless oil; [α]_D_^23^ +7.26 (*c* = 0.27, MeOH); IR (KBr) ν_max_ cm^−1^: 2956, 2868, 1738, 1717, 1457, 1407, 1368, 1262, 1158, 1079; ^1^H and ^13^C NMR data, see Table 1 and Table 2; HRESIMS (*m/z*): 259.1674 [M + Na]^+^ (calcd. for C_15_H_24_O_2_Na, 259.1674); CD λ_max_ nm (Δε): 212 (−2.8).

Sinularone B (**2**): obtained as colorless oil; [α]_D_^23^ +0.60 (*c* = 0.43, MeOH); IR (KBr) ν_max_ cm^−1^: 3489, 2957, 2868, 1737, 1707, 1653, 1377, 1326, 1046; ^1^H and ^13^C NMR data, see Table 1 and Table 2; HRESIMS (*m/z*): 305.1732 [M + Na]^+^ (calcd. for C_16_H_26_O_4_Na, 305.1729); CD λ_max_ nm (Δε): 210 (+3.6).

Sinularone C (**3**): obtained as colorless oil; [α]_D_^23^ +2.20 (*c* = 0.14, MeOH); IR (KBr) ν_max_ cm^−1^: 2954, 2920, 2863, 1730, 1710, 1654, 1458, 1377, 1164, 1062; ^1^H and ^13^C NMR data, see Table 1 and Table 2; HRESIMS (*m/z*): 259.1315 [M + Na]^+^ (calcd. for C_16_H_26_O_4_Na, 259.1310); CD λ_max_ nm (Δε): 203 (+5.0).

Sinularone D (**4**): obtained as colorless oil; [α]_D_^23^ −3.22 (*c* = 0.09, MeOH); IR (KBr) ν_max_ cm^−1^: 2958, 2926, 2872, 1778, 1710, 1657, 1458, 1378, 1165; ^1^H and ^13^C NMR data, see Table 1 and Table 2; HRESIMS (*m/z*): 259.1313 [M + Na]^+^ (calcd. for C_16_H_26_O_4_Na, 259.1310); CD λ_max_ nm (Δε): 222 (−1.3).

Sinularone E (**5**): obtained as colorless oil; [α]_D_^23^ +18.0 (*c* = 0.04, MeOH); IR (KBr) ν_max_ cm^−1^: 3422, 2959, 2854, 1735, 1629, 1458, 1377, 1165; ^1^H and ^13^C NMR data, see Table 1 and Table 2; HRESIMS (*m/z*): 275.1612 [M + Na]^+^ (calcd. for C_1__5_H_2__4_O_3_Na, 275.1623); CD λ_max_ nm (Δε): 221 (+5.59).

Sinularone F (**6**): obtained as colorless oil; [α]_D_^23^ +22.6 (*c* = 0.03, MeOH); IR (KBr) ν_max_ cm^−1^: 3420, 2958, 2856, 1730, 1612, 1459, 1377, 1120; ^1^H and ^13^C NMR data, see Table 1 and Table 2; HRESIMS (*m/z*): 275.1610 [M + Na]^+^ (calcd. for C_1__5_H_2__4_O_3_Na, 275.1623); CD λ_max_ nm (Δε): 219 (−2.56).

Sinularone G (**7**): obtained as colorless oil; [α]_D_^23^ +4.03 (*c* = 0.10, MeOH); IR (KBr) ν_max_ cm^−1^: 3446, 2958, 2921, 1730, 1633, 1457, 1376, 1163; ^1^H and ^13^C NMR data, see Table 1 and Table 2; HRESIMS (*m/z*): 251.0886 [M + Na]^+^ (calcd. for C_11_H_16_O_5_Na, 251.0895); CD λ_max_ nm (Δε): 213(+1.21), 245 (+0.31).

Sinularone H (**8**): obtained as colorless oil; [α]_D_^23^ +3.70 (*c* = 0.12, MeOH); IR (KBr) ν_max_ cm^−1^: 3445, 2958, 2924, 1725, 1630, 1456, 1376, 1165; ^1^H and ^13^C NMR data, see Table 1 and Table 2; HRESIMS (*m/z*): 265.1041 [M + Na]^+^ (calcd. for C_12_H_18_O_5_Na, 265.1052); CD λ_max_ nm (Δε): 213 (+0.25), 230 (−0.01).

Sinularone I (**9**): obtained as colorless oil; [α]_D_^23^ +5.44 (*c* 0.18, MeOH); IR (KBr) ν_max_ 3432, 2959, 2923, 1734, 1633, 1458, 1376, 1162 cm^−1^ : ^1^H NMR (600 MHz, DMSO-*d*_6_) *δ*: 1.67 (1H, m, H-5a), 1.86 (1H, m, H-5b), 1.03 (1H, m, H-6a), 1.12 (1H, m, H-6b), 1.22 (14H, H_2_-7-H_2_-14), 1.50 (2H, m, H_2_-15), 2.25 (2H, t, *J* = 7.4 Hz, H_2_-16), 1.70 (3H, s, H_3_-18), 1.85 (3H, s, H_3_-19), 4.04 (2H, q, *J* = 7.1 Hz, Me-CH_2_), 1.17 (3H, t, *J* = 7.1 Hz); ^13^C NMR (150 MHz, DMSO-*d*_6_) *δ*: 172.1 (C, C-1), 123.9 (C, C-2), 158.9 (C, C-3), 107.7 (C, C-4), 36.1 (CH_2_, C-5), 23.0 (CH_2_, C-6), 29.3 (CH_2_, C-7), 29.2 (CH_2_, C-8), 29.1 (CH_2_, C-9), 28.8 (CH_2_, C-10), 29.1 (CH_2_, C-11), 29.2 (CH_2_, C-12), 29.2 (CH_2_, C-13), 29.1 (CH_2_, C-14), 24.9 (CH_2_, C-15), 33.9 (CH_2_, C-16), 173.2 (C, C-17), 8.5 (CH_3_, C-18), 11.0 (CH_3_, C-19), 14.1 (CH_3_), 60.0 (CH_2_); HRESIMS (*m/z*): 391.2456 [M + Na]^+^ (calcd. for C_21_H_36_O_5_Na, 391.2460); CD λ_max_ nm (Δε):213 (+1.25), 221 (−0.02), 244 (−1.10).

## 4. Conclusions

The *Sinularia* genus commonly produces cembranoids, which are considered to be the biomarkers for chemotaxonomy. The structural patterns such as cyclopentenone/cyclopentanone and unsaturated γ-lactone are unusual natural products found from the soft coral genus *Sinularia*. Present work provided a group of new cyclopentanone/cyclopanone and butenolide-type analogues to enrich the chemical diversity of *Sinularia* corals. The unprecedented 2,3-dimethylbutenolide based natural products were originally found from marine red algae [24] and brittle star [17], implying that dimethylbutenolides may be the signal molecules of the soft coral specimen to maintain coexistence with other benthos. This is the first report to indicate derivatives of unsaturated γ-lactone possessing potent antifouling activity. The metabolites containing a cyclopentenone or cyclopentanone ring are rarely discovered in marine soft corals, but they are often obtained from marine microorganisms. In addition, dimethylbutenolides showed potent antifouling activities, indicating that they contribute to a chemical ecological function. The literature survey revealed that the cyclopentenone group is relevant for cytotoxicity [25], thus cyclopentenone containing metabolites are regarded as the cytotoxins involved in chemical defense against predators. Whether the butenolides or cyclopentenones originated from a coral host or derived from other benthos serving as a food chain are uncertain. 

The ethoxylated compounds such as **2**, **7**–**9** are probably artifacts derived during the extraction process, whereas the naturally occurring forms are suggested to be free of the ethyl group.
